# Reverse Engineering and Robotics as Tools for Analyzing Neural Circuits

**DOI:** 10.3389/fnbot.2020.578803

**Published:** 2021-01-26

**Authors:** Ioannis Pisokas

**Affiliations:** Institute of Perception, Action and Behaviour, School of Informatics, University of Edinburgh, Edinburgh, United Kingdom

**Keywords:** robotics, neurorobotics, navigation, head direction cells, ring attractor, insect, central complex, Drosophila melanogaster

## Abstract

Understanding neuronal circuits that have evolved over millions of years to control adaptive behavior may provide us with alternative solutions to problems in robotics. Recently developed genetic tools allow us to study the connectivity and function of the insect nervous system at the single neuron level. However, neuronal circuits are complex, so the question remains, can we unravel the complex neuronal connectivity to understand the principles of the computations it embodies? Here, I illustrate the plausibility of incorporating reverse engineering to analyze part of the central complex, an insect brain structure essential for navigation behaviors such as maintaining a specific compass heading and path integration. I demonstrate that the combination of reverse engineering with simulations allows the study of both the structure and function of the underlying circuit, an approach that augments our understanding of both the computation performed by the neuronal circuit and the role of its components.

## 1. Introduction

Neurorobotics attempts to derive inspiration from neuroscience on how the brain solves problems in order to develop robust and adaptive artificial agents. The combination of neuroscience with embodied robot agents provides a platform for testing hypotheses and deciphering the principles on which the brain operates. One approach for deciphering the principles of neuronal circuit operation is to implement phenomenological computational models of the neuronal circuit and then identify and analyze similarities between the models and the neuronal circuit in the hope of learning about the neuronal circuit's architecture. Such an approach is exemplified by work comparing features learned by deep convolutional neural networks with those found in the ventral visual system of animals (e.g., Yamins et al., 2014; Cichy et al., 2016; Yamins and DiCarlo, 2016). Phenomenological models attempt to reproduce the mapping of inputs to outputs while being only weakly constrained with respect to the actual neuronal circuit's architecture, thus admitting a range of possible implementations. Therefore, this approach has the potential to provide inspiration for hypothesis formulation and for focusing further research but does not unravel the actual neuronal circuits of biological organisms.

Another approach for analyzing neuronal circuits is to simulate part of the connectome in order to study the circuit's function. This approach is faithful to the actual neuronal connectivity, thus imposing strong constraints with respect to the biological architecture (as done for example by Kakaria and de Bivort, 2017). This approach has the potential to provide insights about the computation performed by the actual neuronal circuit; however, it does so based on phenomenological observations about computation at the system level and does not provide us with a real mechanistic understanding of the underlying neuronal circuit structure and component interaction.

A third approach is to reverse engineer the actual neuronal circuit in order to decipher its organization and structure. Reverse engineering is a technique traditionally used for unraveling the inner workings of hardware devices (Rekoff, 1985). It aims to describe a system at the component level and explain how its components interact with each other. Once the structure of a neuronal circuit is reverse engineered, we can study how its neurons interact and draw hypotheses about the circuit's function on the basis of its neuronal components, thereby offering a mechanistic level of understanding.

Each of the three approaches has merits on its own, but their combination can provide an even more powerful tool for deciphering the function of neuronal circuits. A component-level understanding of the neuronal circuit structure through reverse engineering can be combined with the second approach, that is, computational simulations in order to understand the circuit's function. Deriving such a mechanistic understanding of the neuronal circuit at the neuron level will enable us to modify and customize it for use in specific applications, including robotics. I present here an example of this approach by reverse engineering the head direction circuit of the fruit fly and then utilizing simulations of a situated robotic agent to characterize the circuit's performance.

### 1.1. Insects as an Example Organism

A limiting factor in the study of any system, including the brain, is the level of detail at which it can be scrutinized. However, where detail is available, understanding structure and function may be difficult because naturally evolved neural systems do not obey an overarching structural simplicity principle. On an interesting crossroad of complexity and available neuroanatomical detail are insects. Insects have relatively small and simple brains compared with vertebrates and yet solve many similar problems, such as perception, navigation, foraging, homing, and reproduction. Recent developments of genetic tools and methods provide us with the unique opportunity to study insect brains at the single neuron level. The relative simplicity, together with the fine level of detail available about insect brains, enable us to reverse engineer their neuronal circuits, understand their operation and derive principles that can guide our design of solutions to problems in robotics.

Recent research in insect neurobiology has focused on the study of the central complex of the fruit fly *Drosophila melanogaster*. The central complex is a brain structure that has been preserved through millions of years of evolution and exists across all insect species (Homberg et al., 2011). This brain structure has been implicated in spatial orientation (Neuser et al., 2008; Triphan et al., 2010; Homberg et al., 2011), locomotor control (Strauss, 2002; Ritzmann et al., 2012; Martin et al., 2015; Varga et al., 2017), visual memory (Liu et al., 2006; Neuser et al., 2008; Ofstad et al., 2011), and path integration (Cope et al., 2017; Stone et al., 2017). The central complex consists of five neural formations: the protocerebral bridge, the ellipsoid body, the fan shaped body, the noduli, and the asymmetric bodies (Wolff and Rubin, 2018). The neuronal connectivity of the central complex has an intricate and yet topographically regular structure. Tracing the neurons of the whole central complex is still an ongoing task; however, most of the neurons innervating two of its structures, the protocerebral bridge (PB) and the ellipsoid body (EB), have been traced in adequate detail in the fruit fly *D. melanogaster*, by multiple labs (e.g., Green and Maimon, 2018; Wolff and Rubin, 2018; Turner-Evans et al., 2020), allowing us to reverse engineer the underlying circuit.

Calcium imaging of the neurons that innervate both the PB and the EB, while a tethered fruit fly is walking or flying in a virtual reality environment, has revealed a striking relationship between neuronal activity and behavior. Specifically, it has been observed that the neuronal ensemble maintains localized spiking activity—commonly called an activity “bump”—that moves from one group of neurons to the next as the animal rotates with respect to its surroundings (Seelig and Jayaraman, 2015; Kim et al., 2017; Giraldo et al., 2018). The neuronal activity “bump” is maintained even when the visual stimulus is removed, and it moves relative to the no longer visible cue as the animal walks in darkness (Seelig and Jayaraman, 2015). Thus, this neuronal activity appears to constitute an internal encoding of heading, which is strongly reminiscent of the hypothetical ring attractor (Amari, 1977) proposed by Skaggs et al. (1995) to account for the “head direction” cells of rats (Taube et al., 1990).

Ring attractor models typically consist of a topological ring of neurons utilizing opposing excitatory and inhibitory synapses to establish a unique activity “bump” around the ring, with neurons forming lateral excitatory connections to neighboring neuronal units and inhibitory connections inhibiting neurons on the opposite side of the ring (Taube et al., 1990; Skaggs et al., 1995; Zhang, 1996). The result is that the most active neurons suppress the activity of all other neurons around the ring and a unique “bump” of activity emerges. Adequate external stimulation of a neuron in the ring causes the activity “bump” to move to the new most active neuron and this new attractor state to be maintained even after the stimulus is removed. This type of ring attractor model can reproduce the phenomena recorded via calcium imaging of fruit flies (Kim et al., 2017). However, this is only a phenomenological similarity and does not reveal whether the actual neuronal circuit in the animal's brain has the same form as this hypothetical ring attractor or if a different circuit structure produces the phenomena.

In this paper, I investigate the circuit structure and function separately. I illustrate that using reverse engineering on the projection patterns of the fruit fly's heading tracking neuronal circuit is possible to reveal an underlying connectivity that has a ring structure with eight-fold radial symmetry. I subsequently illustrate that combining insights from reverse engineering with simulations allows us to explore the circuit's function and identify some notable differences from classic ring attractor models, which may contribute to the stability and flexibility of its function.

## 2. Neuronal Circuit Analysis

As an illustrative example of the usefulness of reverse engineering of a neuronal circuit, I will present a detailed explanation of the process applied to the fruit fly's head tracking circuit. This technique was recently applied to two insect species and the results were presented in Pisokas et al. (2020). Here, I illustrate the reverse engineering process in detail to enable others to apply it to different neuronal circuits and I show that this approach can help us understand neuronal circuit structure and function.

The circuit structure will be reverse engineered at the neuron level of abstraction, removing details about neuron anatomy, biophysics, and location. In the particular case of the central complex, neurons follow a topographically regular pattern, which offers an advantage that will be exploited in the process. The reverse engineering procedure described in the sequel consists of three steps:

1. First, we identify neuron classes. Each neuron class follows a particular connectivity pattern.
2. Second, we identify the neural volumes where neurons form synapses with each other. We number these volumes so that we can systematically inspect them.
3. Third, for each class of neurons, we record connections between neurons in a directed graph. To this end, we focus on each neuron in turn and add its output connections with other neurons.

In the central complex, there is redundancy in the neuronal circuit due to the mirrored connectivity in the left and right hemispheres. The final graphs shown here have eight neurons for each neuron class, which is the result of an iterative process removing redundancy in each iteration. In the first iteration, there were as many graph nodes as there are neurons in the circuit. In each iteration, duplicate neurons were removed and the same process was repeated to reach the final result.

### 2.1. What Is the Effective Neuronal Circuit Structure?

A subset of neuron types in the central complex appear to be the key elements of a circuit with a ring structure. The connectivity of the neurons has been inferred here from anatomical data, with overlapping neuronal terminals assumed to form synapses between them (Wolff et al., 2015; Wolff and Rubin, 2018). The following analysis considers four types of neurons, the E-PG, P-EG, P-EN, and Delta7 neurons (Table 1), in accordance with previous work (Green et al., 2017; Kakaria and de Bivort, 2017; Kim et al., 2017; Su et al., 2017). Each of the four types of neurons follows a particular connectivity pattern.

**Table 1:** Neuronal nomenclature.

**Model neuron name**	**Included neurons**	**Systematic names (Wolff and Rubin, 2018)**
E-PG	E-PG and E-PG_*T*_	PBG1–8.b-EBw.s-D/V GA.b and PBG9.b-EB.P.s-GA-t.b
P-EN	P-EN	PBG2-9.s-EBt.b-NO1.b
P-EG	P-EG	PBG1–9.s-EBt.b-D/V GA.b
Delta7	Delta7 or Δ7	PB18.s-GxΔ7Gy.b and PB18.s-9i1i8c.b

*Correspondence between neuron names used in the model and the neurons names used in the literature. The first column shows the names used in this paper to refer to each group of neurons. The other two columns provide the shorthand consensus names and the full neuron names used in the literature*.

**Figure 1:**
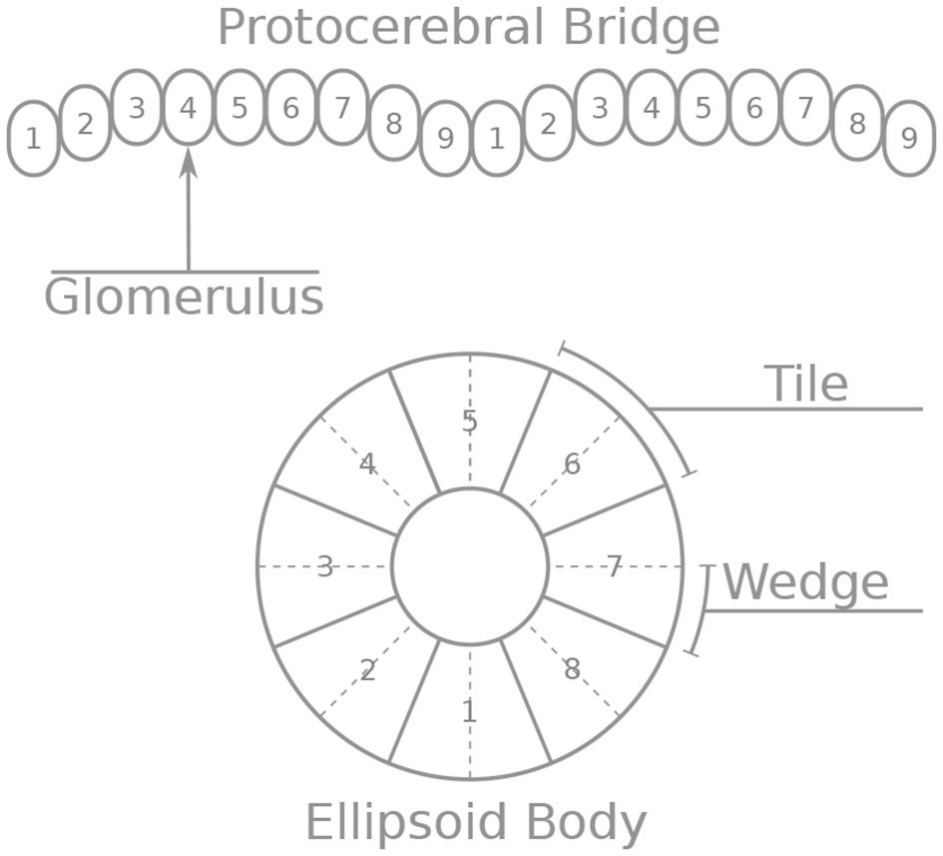
Schematic depiction of the protocerebral bridge and ellipsoid body anatomy. The protocerebral bridge (PB) consists of nine “glomeruli” in each hemisphere, arranged the one next to the other. The ellipsoid body (EB) consists of eight sectors called “tiles” further divided into “wedges”.

These neurons innervate two of the central complex structures: the protocerebral bridge and the ellipsoid body. The protocerebral bridge (PB) consists of nine “glomeruli” in each hemisphere, arranged the one next to the other (Figure 1). The ellipsoid body (EB) consists of eight sectors called “tiles.” Each tile is further divided into two “wedges” (Figure 1). Neurons form synapses within glomeruli of the PB or tiles of the EB. Since all neurons considered here form synapses in the PB, we number the neurons by the glomerulus they innervate. Since Delta7 neurons have both their input and output terminals in the PB we number them by the glomerulus where their output terminals are located.

The E-PG, P-EG, and P-EN neurons are assumed to have excitatory effect on their postsynaptic neurons, while Delta7 neurons are assumed to form inhibitory synapses with their postsynaptic neurons, as Kakaria and de Bivort (2017) proposed. These assumptions are consistent with RNA sequencing, indicating that E-PG, P-EG, and P-EN are cholinergic while Delta7 glutamatergic (Turner-Evans et al., 2020). At this point, we have done the preparatory work (steps 1 and 2) and we can proceed with deriving the underlying effective circuit by redrawing the connectivity as a directed graph, which is a convenient representation for studying circuit topology.

#### 2.1.1. Inhibitory Circuit

**Figure 2:**
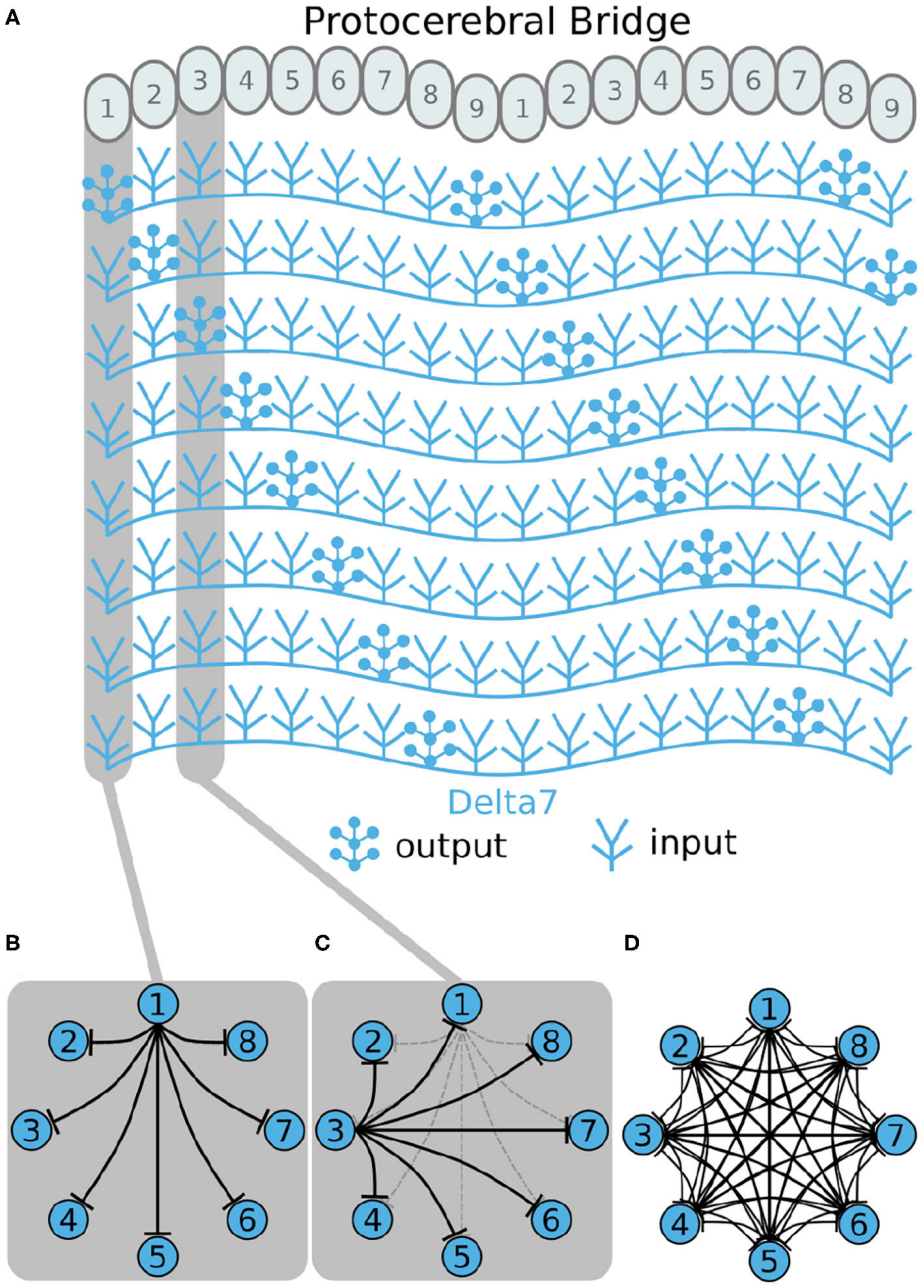
Effective connectivity of the inhibitory (Delta7) neurons. **(A)** Schematic depiction of how the eight Delta7 neuron types innervate the glomeruli of the protocerebral bridge. **(B,C)** The effective connectivity in the first and third glomeruli is depicted as directed graphs with discs representing neurons and lines inhibitory synapses between them. **(D)** The effective neuronal circuit connectivity of the eight Delta7 neurons. Each Delta7 neuron inhibits all other Delta7 neurons resulting in an all-to-all inhibition pattern.

First, we will walk through reverse engineering the connectivity of the first class of neurons, the eight inhibitory Delta7 neurons. These neurons innervate the whole length of the PB (Figure 2A). Anatomical evidence shows that each Delta7 neuron has output synaptic terminals in two or three glomeruli along the PB and input terminals across all remaining glomeruli (Wolff and Rubin, 2018). Output terminal domains of each neuron are separated by seven glomeruli (Figure 2A).

Each Delta7 neuron forms synapses with all other Delta7 neurons in two or three glomeruli along the PB (Figure 2A). Starting from the first glomerulus (glomerulus 1) in the left hemisphere, we see that one neuron has output terminals while the other seven neurons have input terminals; we add arrows in the directed graph to indicate which neurons receive input synapses from this first neuron (Figure 2B). This can be systematically repeated for the synapses in each glomerulus from left to right (glomeruli 1–8 in the left hemisphere). Then proceeding to glomerulus 9 and through 1–9 in the right hemisphere, we observe that the same connectivity pattern repeats. Since we are interested only in the effective connectivity, we do not preserve information about repeated connections between neurons in the final directed graph (Figure 2D). As such, the two or three synaptic connections between pairs of Delta7 neurons are reduced to one single connection between each pair of nodes in the simplified effective circuit in Figure 2D. This reduction to the essential connectivity is crucial for gaining an understanding of the circuit structure. The directed graph depiction of the circuit makes it evident that each Delta7 neuron forms synapses with and inhibits all other Delta7 neurons. Therefore, a uniform, all-to-all, inhibition pattern is revealed.

#### 2.1.2. Excitatory Circuit

**Figure 3:**
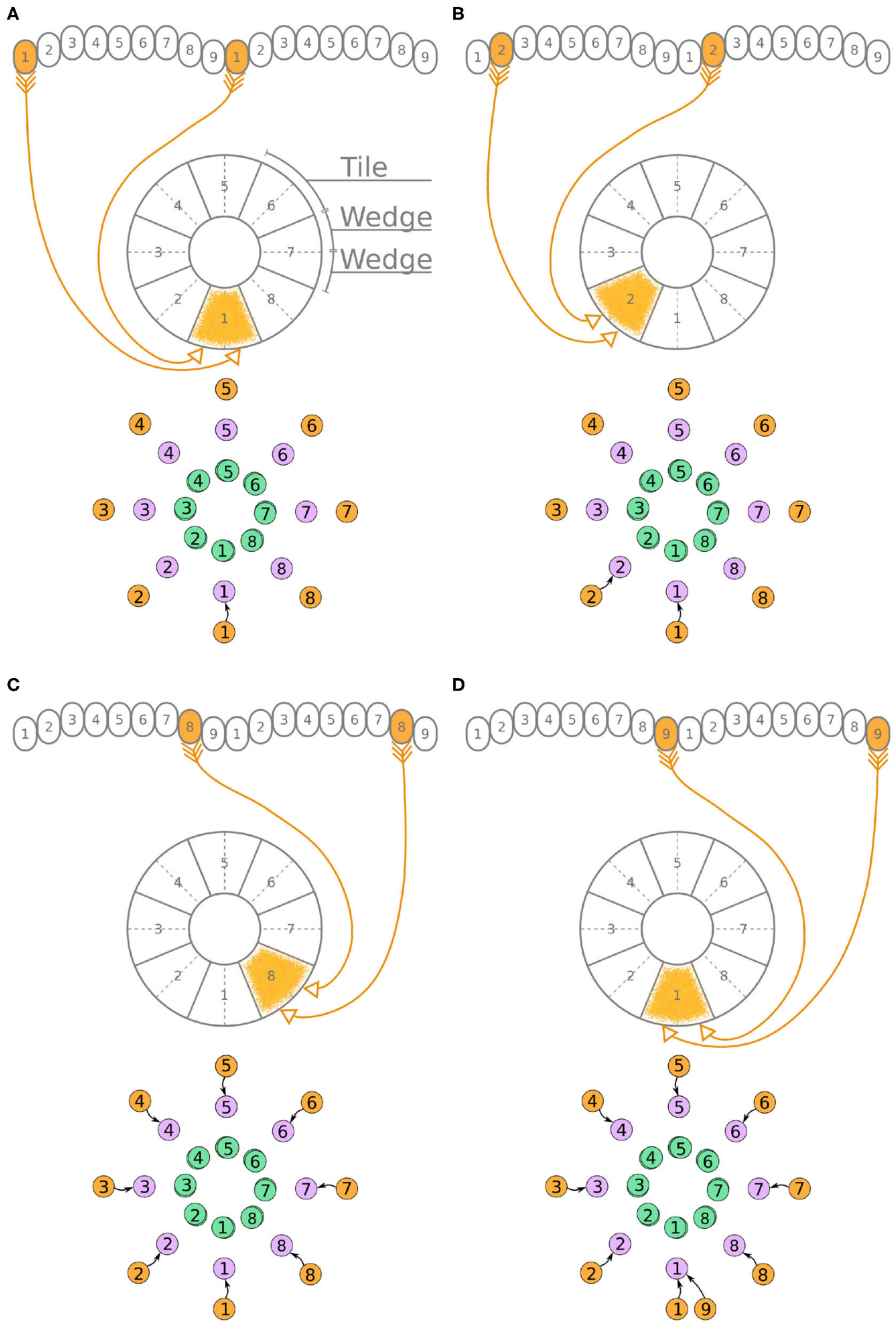
**(A–D)** The connectivity pattern of P-EG neurons of the fruit fly. The top of each panel shows the connectivity pattern of a pair of P-EG neurons with their synaptic domains and connectivity patterns (see main text for detailed description). The bottom of each panel depicts the effective connectivity of the circuit as a directed graph. In the top portion of the panels each arrow represents a neuron. In the bottom portions of the panels, colored discs represent neurons and arrows represent synaptic connections.

**Figure 4:**
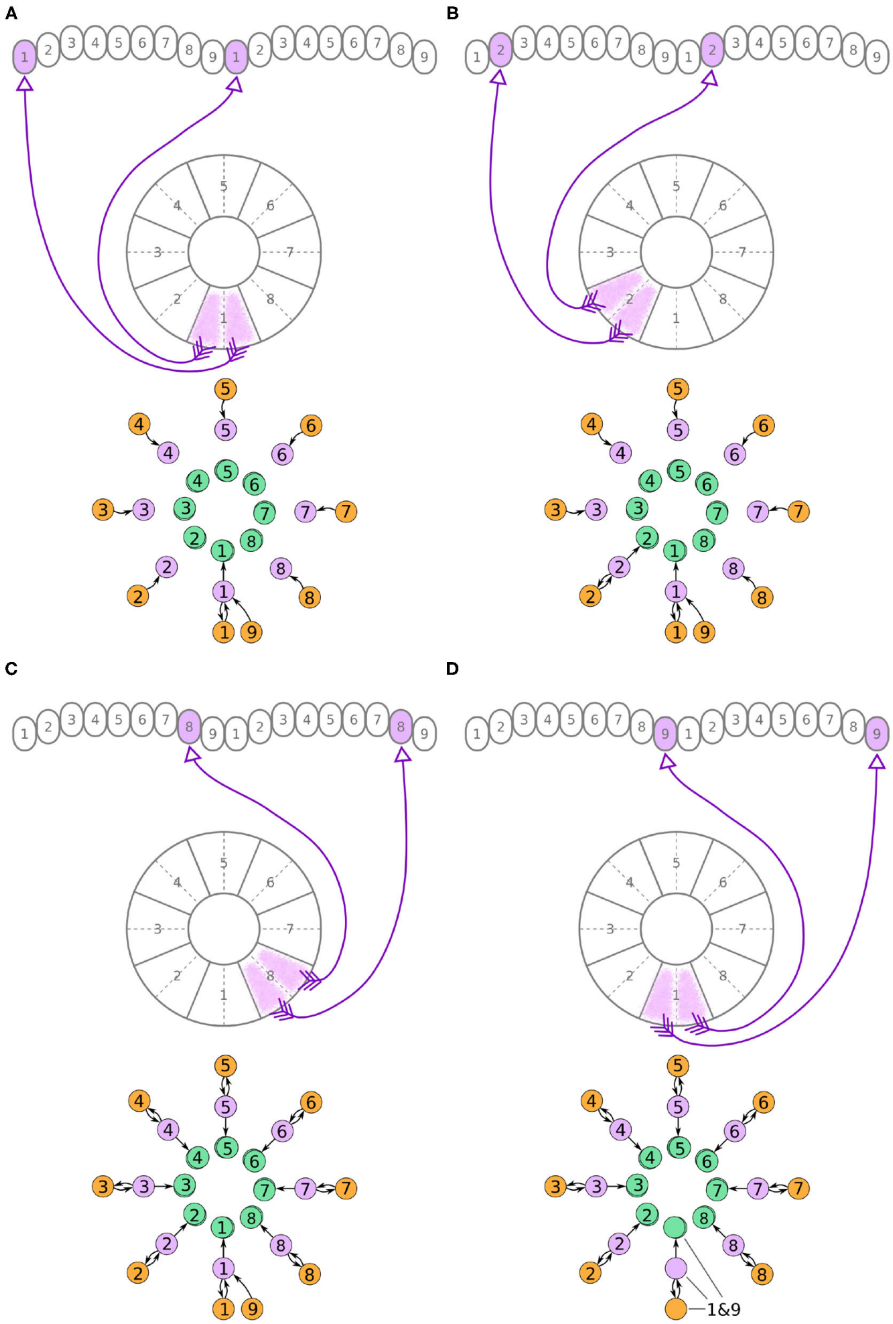
**(A–D)** The connectivity pattern of the E-PG neurons of the fruit fly. The top of each panel shows the connectivity pattern of pairs of E-PG neurons with their synaptic domains and connectivity patterns (see main text for detailed description). The bottom of each panel depicts the effective connectivity of the circuit as a directed graph. In the top portion of the panels each arrow represents a neuron. In the bottom portions of the panels, colored discs represent neurons and arrows represent synaptic connections.

**Figure 5:**
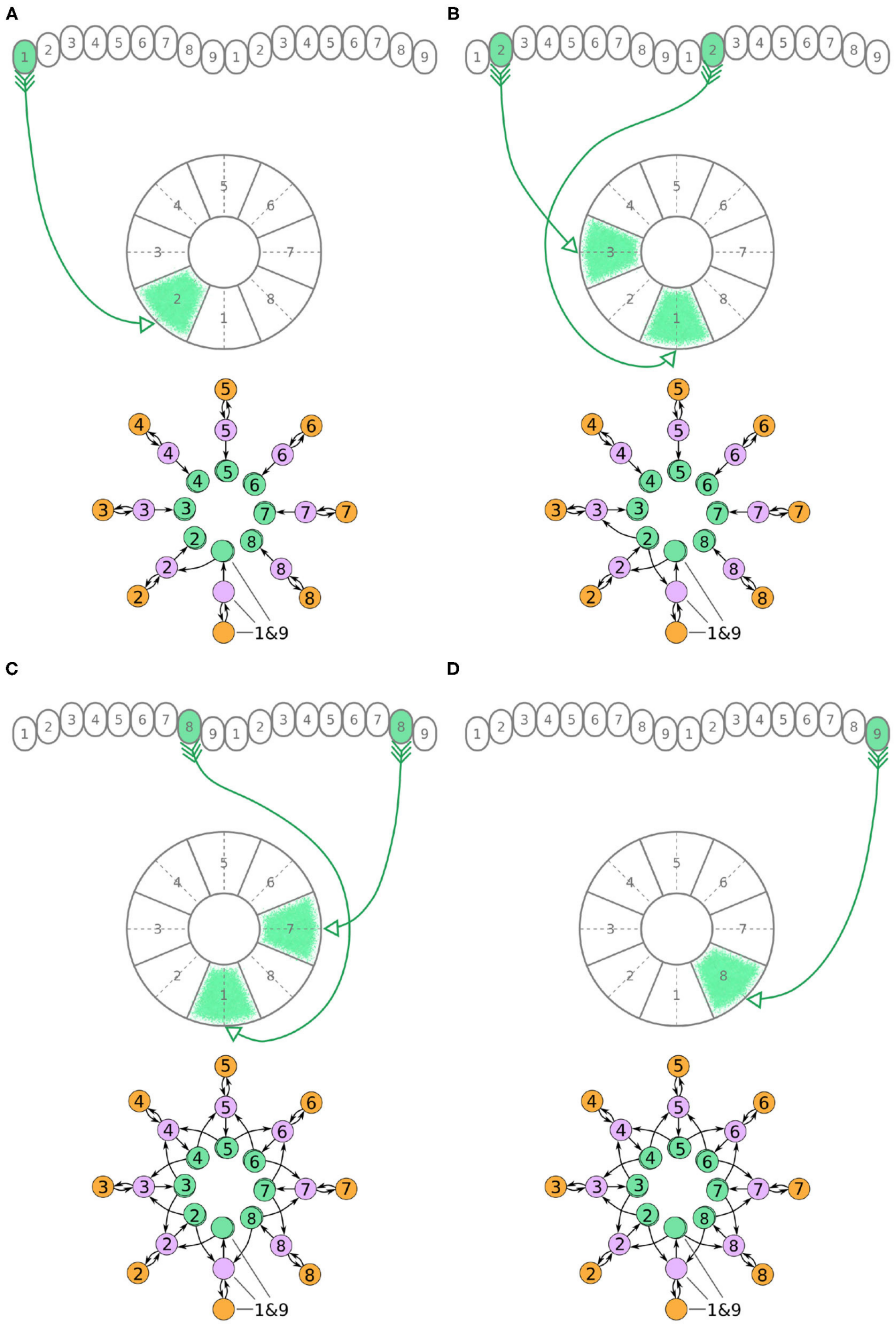
**(A–D)** The connectivity pattern of P-EN neurons of the fruit fly. The top of each panel shows examples of P-EN neurons with their synaptic domains and connectivity patterns (see main text for detailed description). The bottom of each panel depicts the effective connectivity of the circuit as a directed graph. In the top portion of the panels each arrow represents a neuron. In the bottom portions of the panels, colored discs represent neurons and arrows represent synaptic connections.

Now, we will walk through the steps of reverse engineering the excitatory portion of the circuit. The excitatory portion of the circuit consists of three classes of neurons, the P-EG, E-PG, and P-EN neurons. The synaptic terminals of each neuron are confined to one glomerulus of the PB (Figures 3–5). In the EB, the synaptic terminals of E-PG neurons are constrained in single wedges (half tiles) while the synaptic terminals of P-EN and P-EG neurons extend to whole tiles. In our schematic of the anatomy (see Figure 3), the glomeruli are numbered 1–9, left-to-right, in each PB hemisphere, and the EB tiles are numbered 1–8 clockwise. The neurons are numbered by the glomerulus they innervate, e.g., P-EN_1_. For brevity, a tile numbered *n* is denoted as T*n* and a glomerulus numbered *m* as G*m*.

According to calcium and electrophysiology recordings (Turner-Evans et al., 2017), there must be one activity “bump” emerging around the EB and two activity “bumps” along the PB, one in each hemisphere. Preliminary simulation of the neuronal circuit, using the connectivity matrix derived from the neuronal anatomy, confirmed that the two activity “bumps” are centered around neurons innervating identically numbered PB glomeruli. That is, if the one activity “bump” is centered around G5 in the left hemisphere, the second activity “bump” will be centered around G5 in the right hemisphere. This observation about function will be used here in order to simplify the circuit structure and derive the effective connectivity.

Under the aforementioned numbering scheme, each P-EG neuron has synaptic terminals in identically numbered PB glomeruli and EB tiles (Figure 3A). That is, P-EG_1_ has synaptic terminals in tile T1 and glomeruli G1 in both hemispheres of the PB. Since the two P-EG_1_ neurons receive equal input in glomeruli G1, in both hemispheres, and connect to the same EB tile, T1, they are replaced with a single effective functional unit, as shown at the bottom of panel Figure 3A, in the form of a directed graph. The same reasoning can be repeated for the next pair of neurons, P-EG_2_, that connect glomeruli G2 to tile T2 (Figure 3B). Figure 3C shows the resulting effective circuit if these steps are followed all the way until P-EG_8_, the pair of neurons connecting glomeruli G8 to tile T8. Finally, we consider the last pair of neurons, P-EG_9_; this pair of neurons connects glomeruli G9 to tile T1, breaking the pattern. These neurons are represented with a new node in the graph, but as it will become apparent in the next paragraph, the P-EG_9_ neurons receive the same input as P-EG_1_ neurons allowing us to combine them.

A second class of cells, E-PG neurons, also have synaptic terminals in equally numbered EB tiles and PB glomeruli, following a similar pattern with the P-EG neurons but with their input and output terminals on opposite ends (Figure 4). Pairs of these neurons can again be replaced by single equivalent neuronal units because they receive input from the same EB tile and innervate equally numbered glomeruli in both hemispheres. The first pair of E-PG neurons, E-PG_1_, receive input in tile T1 and provide output in glomeruli G1 in both hemispheres (Figure 4A). Adding the corresponding connections results in the directed graph shown at the bottom of Figure 4A. Repeating the same for neurons E-PG_2_ to E-PG_8_ results in the graph shown in Figure 4C. Here, again there is a ninth pair of cells, the E-PG_9_ neurons, that connect T1 to G9 in both hemispheres. These neurons receive the same input signal as E-PG_1_ neurons but provide output to neurons in G9 instead of G1. Therefore, P-EG_1_ and P-EG_9_ neurons receive the same signal, in glomeruli G1 and G9, and provide the same output to both E-PG_1_ and E-PG_9_ neurons, as mentioned in the previous paragraph. This allows us to combine the P-EG_1_ and P-EG_9_ neurons into one single unit in the graph of Figure 4D.

Unlike the P-EG and E-PG neurons, the P-EN neurons do not innervate the two middlemost glomeruli (G9 in the left hemisphere and G1 in the right hemisphere, Wolff et al., 2015). There are, therefore, eight pairs of P-EN neurons, spanning glomeruli 1–8 in the left hemisphere and 2–9 in the right hemisphere. P-EN_2_ through P-EN_8_ form pairs connecting equally numbered glomeruli to two different EB tiles, one shifted to the left and one to the right, i.e., P-EN_2_ would connect glomeruli G2 to tiles T1 and T3 (Figure 5B), P-EN_3_ would connect glomeruli G3 to tiles T2 and T4, etc. P-EN_2_ neurons form synapses with E-PG_1_ neurons in T1 and E-PG_3_ neurons in T3, which would innervate glomeruli G1 and G3, respectively. The exceptions in this pattern are the two P-EN neurons receiving input from the outermost glomeruli of the PB, P-EN_1_ and P-EN_9_. P-EN_1_ is unpaired and connects G1 of the left hemisphere to T2 (Figure 5A). P-EN_9_ is also unpaired and connects G9 of the right hemisphere to T8 (Figure 5D). Since P-EN_1_ and P-EN_9_ receive the same input from E-PG_1_ and E-PG_9_ neurons, they constitute a pair closing the ring, as shown in Figure 5D. In the directed graphs, each pair of P-EN neurons is preserved as two overlapped discs because P-EN neurons not only receive common input in the glomeruli but may also receive differential angular velocity input depending on which PB hemisphere they innervate (Turner-Evans et al., 2017).

It becomes apparent from Figure 5D that the E-PG neurons provide input to the P-EN and P-EG neurons, with P-EG neurons forming recurrent synapses back to E-PG neurons. P-EN neurons provide input to E-PG neurons with a shift of one octant to the left or right.

#### 2.1.3. Overall Circuit

**Figure 6:**
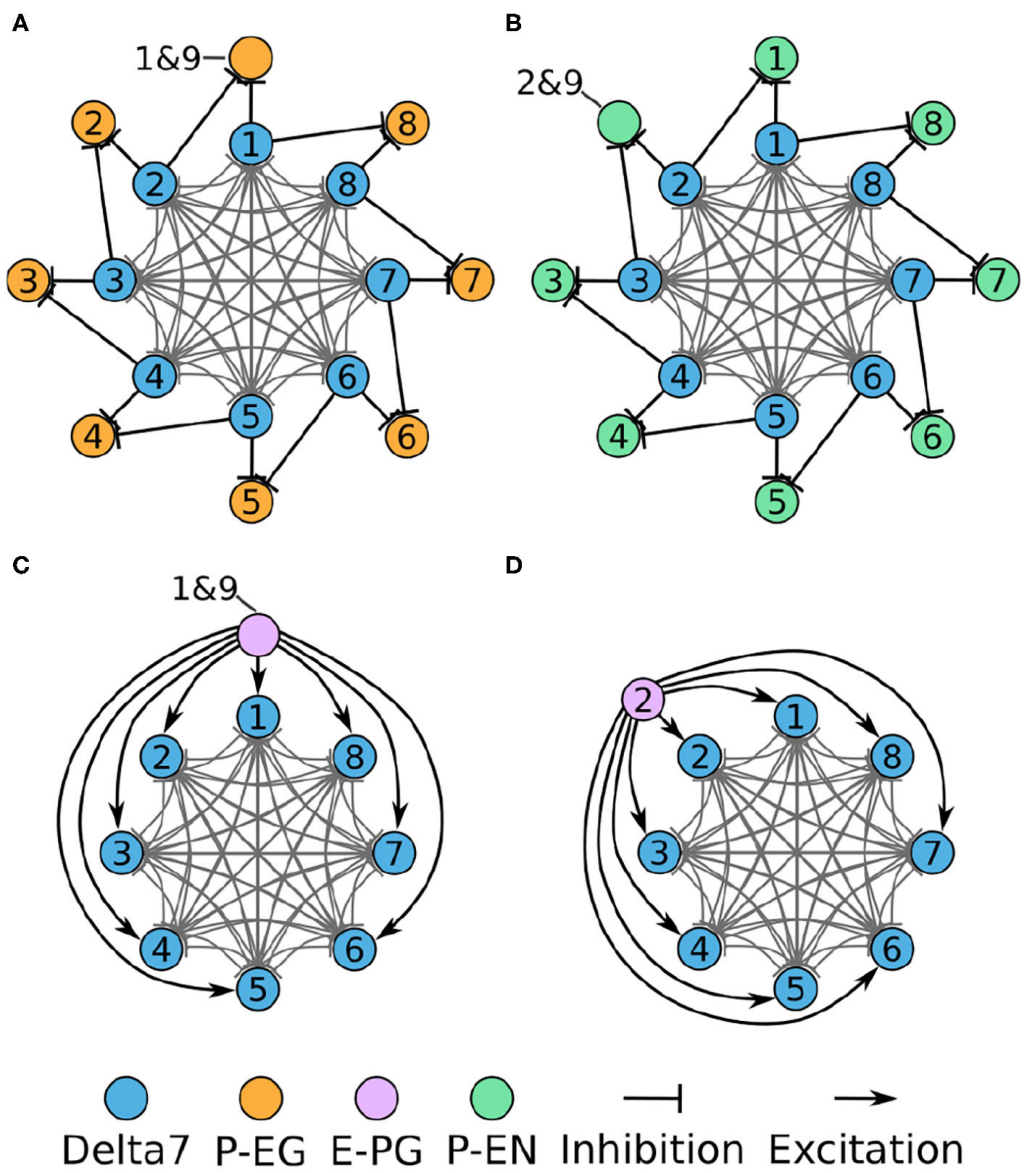
Connectivity of combined excitatory and inhibitory portions of the circuit. Each colored disc represents one or more neurons with the lines representing synaptic connections. **(A)** The connectivity pattern of the Delta7 neurons with the P-EG neurons. **(B)** The connectivity pattern of the Delta7 neurons with the P-EN neurons. **(C,D)** The connectivity pattern of E-PG neurons (E-PG_1&9_ and E-PG_2_). The other E-PG neurons follow the same connectivity pattern rotated around the Delta7 neurons. Each pair of E-PG neurons excites all Delta7 neurons.

In each PB glomerulus, the inhibitory Delta7 neurons form synapses with the three types of excitatory neurons. Figure 6 shows the interaction of the excitatory and inhibitory portions of the circuit. Each Delta7 neuron makes inhibitory synapses to P-EG and P-EN neurons, as well to all other Delta7 neurons (Figures 6A,B). Due to their projection patterns, the Delta7 neurons provide uniform inhibition to all eight octants of the circuit, while E-PG neurons provide input to all Delta7 neurons (Figures 6C,D). For drawing the graphs in Figure 6, Figures 2A, 3–5 were revisited and the connections within each glomerulus were added in the graphs.

The resulting directed graph representation removed the details about the anatomical organization of the EB and the PB while preserving the effective connectivity of the circuit. This analysis revealed that even though the PB is organized in nine glomeruli in each hemisphere, the effective circuit has an eight-fold radial symmetry. This is because the E-PG and P-EG neurons innervating the PB glomeruli G1 and G9, in both hemispheres, have synaptic domains in the same EB tile, T1. This aggregation of synaptic connections between the edges of the PB and T1, results in the closing of the ring between octants 1 and 8 (Figure 5D). The ring topology of the circuit reveals the interaction between components and is indicative of its function.

### 2.2. Computational Model

**Figure 7:**
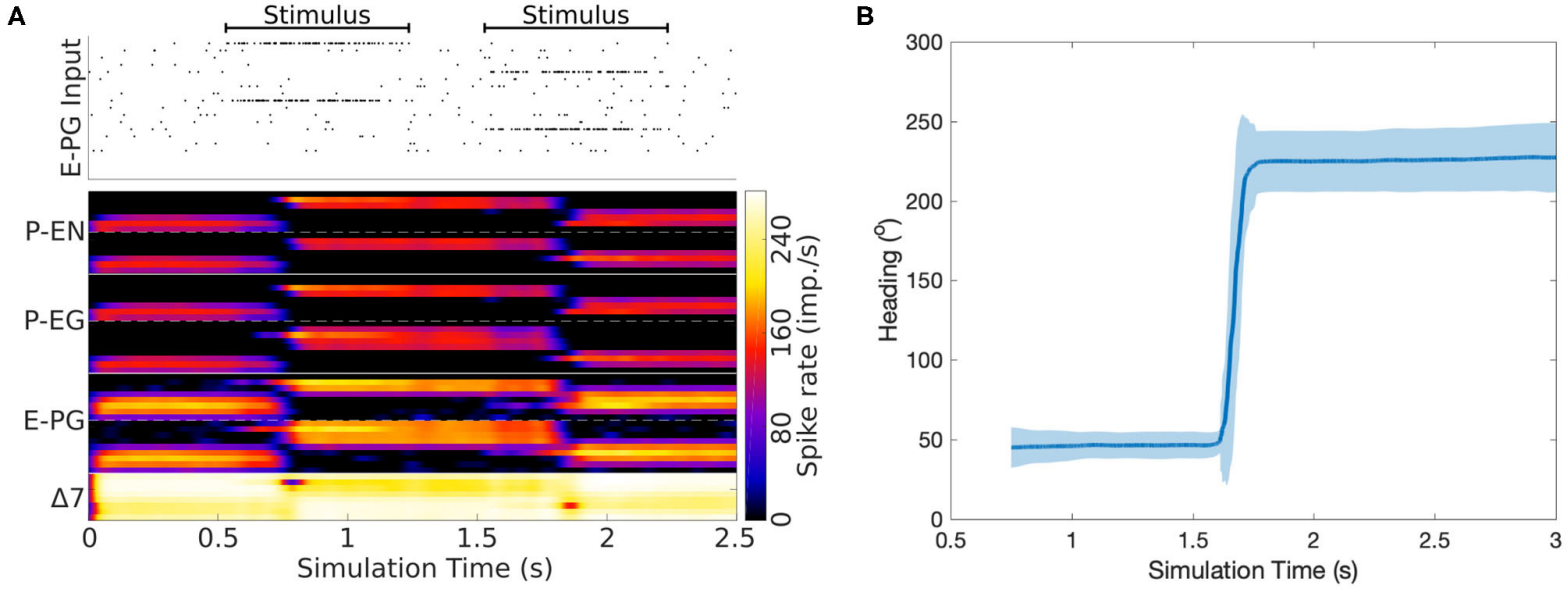
Response of the ring attractor to abrupt changes of stimulus azimuth. **(A)** The spike raster plot on top shows the stimulus provided to the E-PG neurons. The lower part of the plot shows in color coding the spiking rate activity of each neuron in the circuit. At 0.5 s an incoming stimulus sets the initial attractor state of the ring attractor. A “darkness” period of no stimulus follows, during which the “bump” of activity is maintained at the same location. Then a second stimulus, corresponding to a sudden change of heading by 180^*o*^, is provided, producing a sudden change in the position of the “bump,” with this new location then maintained after the stimulus is removed. The order of recorded neurons is the same as shown in the connectivity matrix (Figure 12). **(B)** The mean activity “bump” heading and corresponding standard deviation across time when the ring attractor is stimulated with a step change of heading (80 trials).

Now that we have reverse engineered the circuit structure, we can use simulations to investigate its function and corroborate the role of its components. To this end, a spiking neuron model of the derived circuit was implemented using the connectivity matrix and utilizing leaky integrate and fire neuron models with refractory period (section 4). Since neurophysiological evidence suggests a ring attractor resembling function and the effective circuit structure has the topology and necessary elements for a ring attractor, it was decided to impose the constraint that the circuit should function as a ring attractor. Using this constraint, an optimization algorithm was used to search for synaptic weights that result in a working ring attractor (section 4). The activity “bump” location was set by a heading stimulus provided as incoming spiking activity directly to the E-PG neurons, corresponding to input from Ring neurons (Young and Armstrong, 2010). This heading input mapped the position of a visual cue, or retinotopic landmark position (Seelig and Jayaraman, 2015), around the animal to higher firing rates of E-PG neurons in the corresponding tile of the EB. The neuronal parameters were set to values consistent with evidence from measurements in *D. melanogaster*, as described in section 4. Figure 7 shows examples of neuronal activity in the simulated ring attractor circuit with the activity “bump” transitioning from one attractor state to another in response to a change of the stimulus azimuth.

### 2.3. Situated Agent Behavior

The stimulus used in the preceding simulation was a step function of time, but a real fruit fly or robot would not perform instantaneous turns between heading directions; instead, they would exhibit smoother transitions between headings and a generally variable angular velocity over time. It is, therefore, important to characterize the circuit's performance in such a more natural scenario. For this reason, the flight trajectory of a real fruit fly was next used to simulate an agent turning with respect to a visual landmark. The fruit fly's heading over time was extracted from such a flight trajectory and was used to generate the time series of headings the agent adopts.

**Figure 8:**
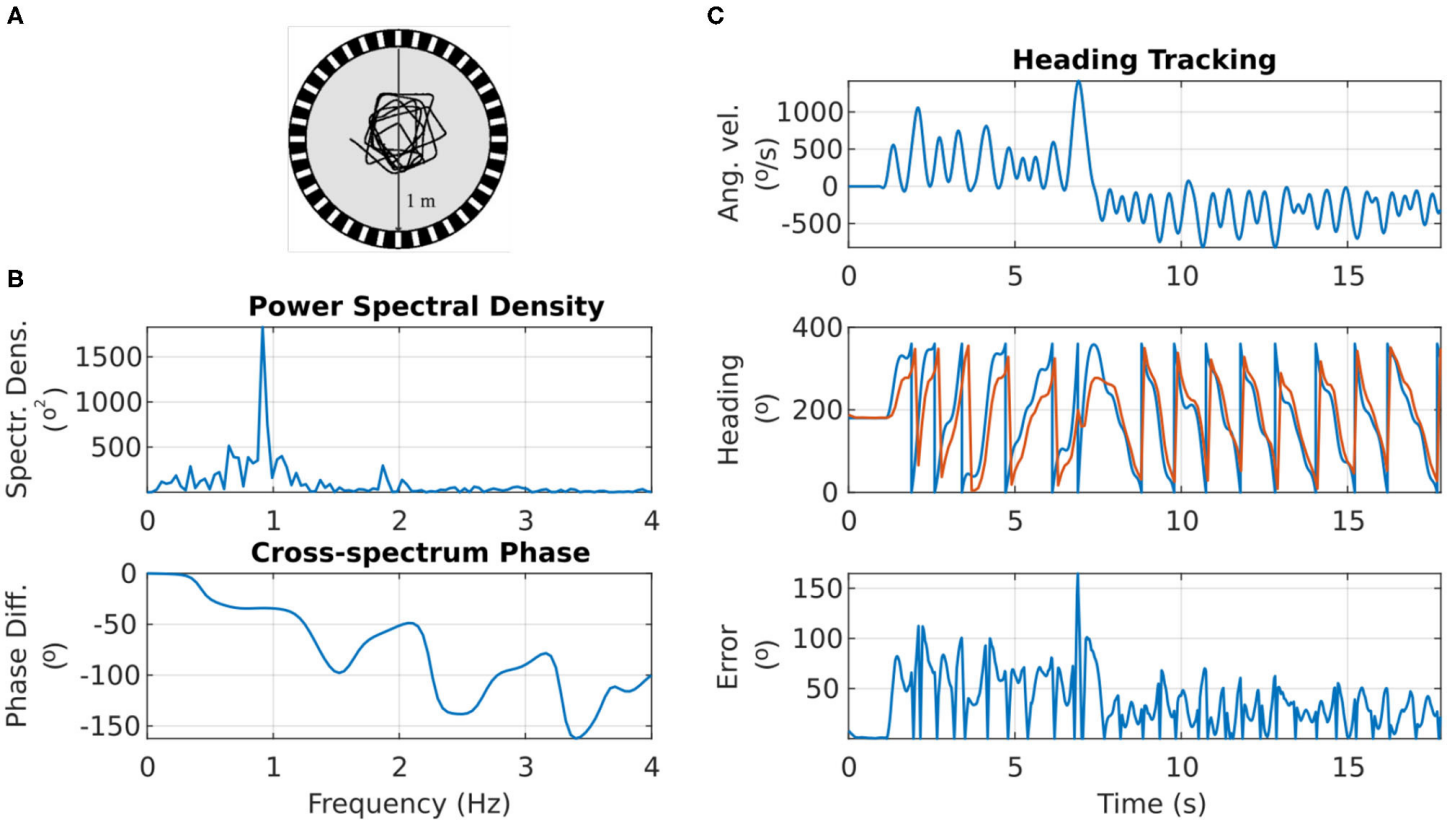
Response of the ring attractor activity “bump” to the heading changes of a real fruit fly. **(A)** Top view of the trajectory of a fruit fly flying in a circular arena (Modified from Tammero and Dickinson, 2002). **(B)** Top: the spectral content of the fruit fly heading progress over time. Bottom: the phase difference between the fruit fly heading and the heading tracked by the ring attractor across frequency components. **(C)** Top: the angular velocity of the fruit fly over time. Middle: the heading of the fruit fly (in blue) and the azimuth of the activity “bump” around the ring attractor (in red). Bottom: the heading error (absolute difference) between the fruit fly heading and the ring attractor tracked heading.

Figure 8A shows the motion trajectory of a fruit fly flying in a circular arena (Tammero and Dickinson, 2002; Figure 2). From the power spectral density plot of the heading over time, we can see that the fruit fly's heading signal has a main period of 1.092 s, corresponding to the fruit fly completing a full rotation around the arena in approximately 1 s (spectral peak at 0.916 Hz in Figure 8B). This was confirmed with calculation of the auto-covariance that produced a mean period of 1.087 s.

The visual landmark's azimuth with respect to the agent was retinotopically mapped to the E-PG neurons around the ring attractor (section 4). The correspondence between the heading of the agent and the heading encoded by the ring attractor circuit is shown in Figure 8C. The ring attractor tracked the agent's heading with an average lag of 100 ms. The exact phase lag depended on the frequency component of the signal, with a trend for higher frequencies—faster heading changes—resulting in increased lag (see bottom plot of Figure 8B). This is an expected effect because neurons have non-zero time constants and response times.

Overall, even though the heading encoded by the ring attractor accumulated error during fast turns of the agent, it caught up with the actual heading as soon as the agent's angular velocity was reduced (Figure 8C). This effect is due to the ring attractor circuit being continually driven by the stimulus' azimuthal position, so if given enough time to respond, the circuit state is readjusted to the stimulus position. It becomes apparent with this situated agent simulation that even though the agent's heading may change faster than the circuit's ability to track it, as soon as the agent slows down, the visual cue input corrects the location of the activity “bump” (Figure 8C).

### 2.4. Role of Circuit Elements

Now that we have both the underlying circuit structure and its computational model, we can draw hypotheses and ask pointed questions about the role of each circuit component. We can artificially manipulate the circuit by removing or replacing functional elements in order to study their effect on circuit function. We recently used this method to investigate the stability of the activity “bump” in the absence of stimulus (Pisokas et al., 2020). We extend this approach here and investigate the circuit's performance as part of a situated agent that turns with respect to a visual cue.

**Figure 9:**
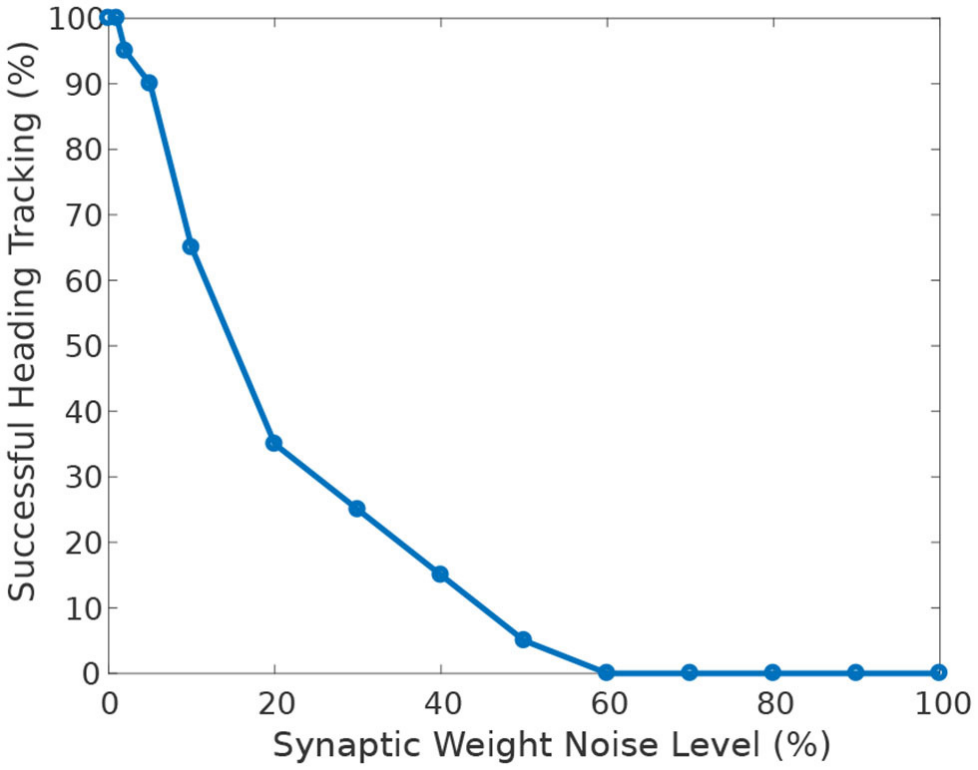
Effect of synaptic weights heterogeneity on heading tracking performance. The ability of the ring attractor to track the heading of a simulated robot, replicating the turns of a real fruit fly, deteriorates as function of synaptic noise. Synaptic noise was introduced by adding values drawn from the Gaussian distribution to the nominal values of all synaptic weights.

Figure 9 shows the effect of heterogeneity of synaptic weights on the ability of the circuit to track the agent's heading when turning with respect to a visual cue. The ability to accurately track the agent's heading deteriorates with increasing heterogeneity (additive Gaussian noise) of synaptic weights.

**Figure 10:**
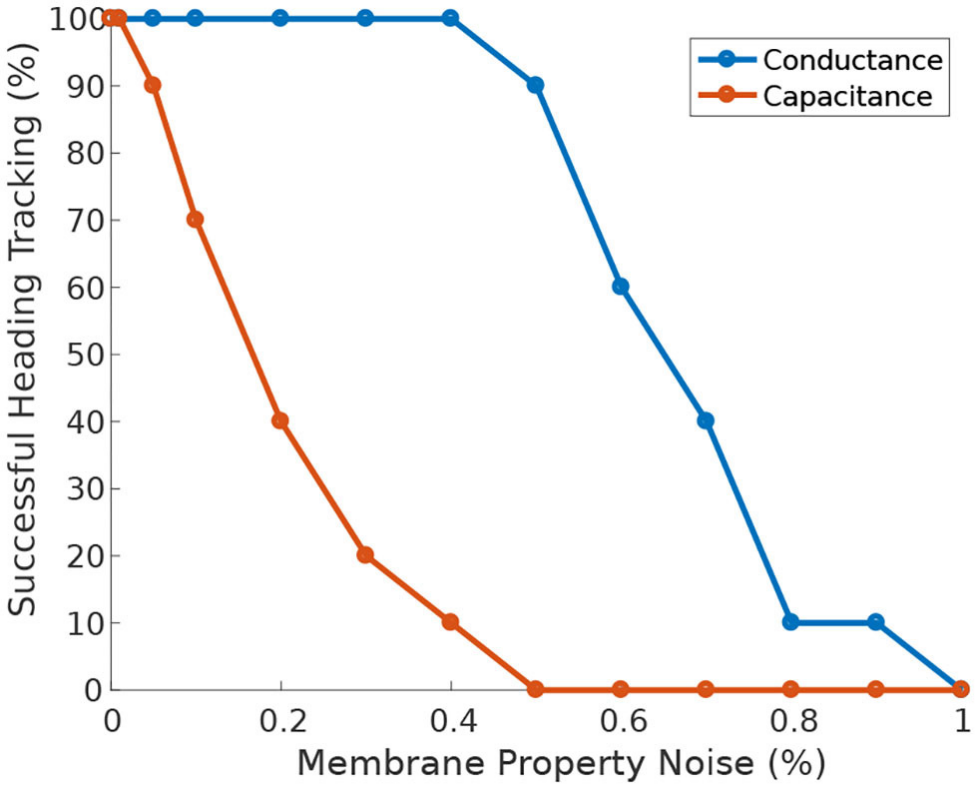
Effect of membrane properties heterogeneity on heading tracking performance. The ability of the ring attractor to track the heading of a simulated robot, replicating the turns of a real fruit fly, deteriorates as function of Gaussian noise added to the neuronal membrane conductance and capacitance.

Furthermore, when the circuit is driven by heading stimulus, it is significantly more tolerant of heterogeneity in neuronal membrane conductance than in membrane capacitance (Figure 10). The circuit can successfully track the agent's heading even when the membrane conductance deviates 50% away from its nominal value.

**Figure 11:**
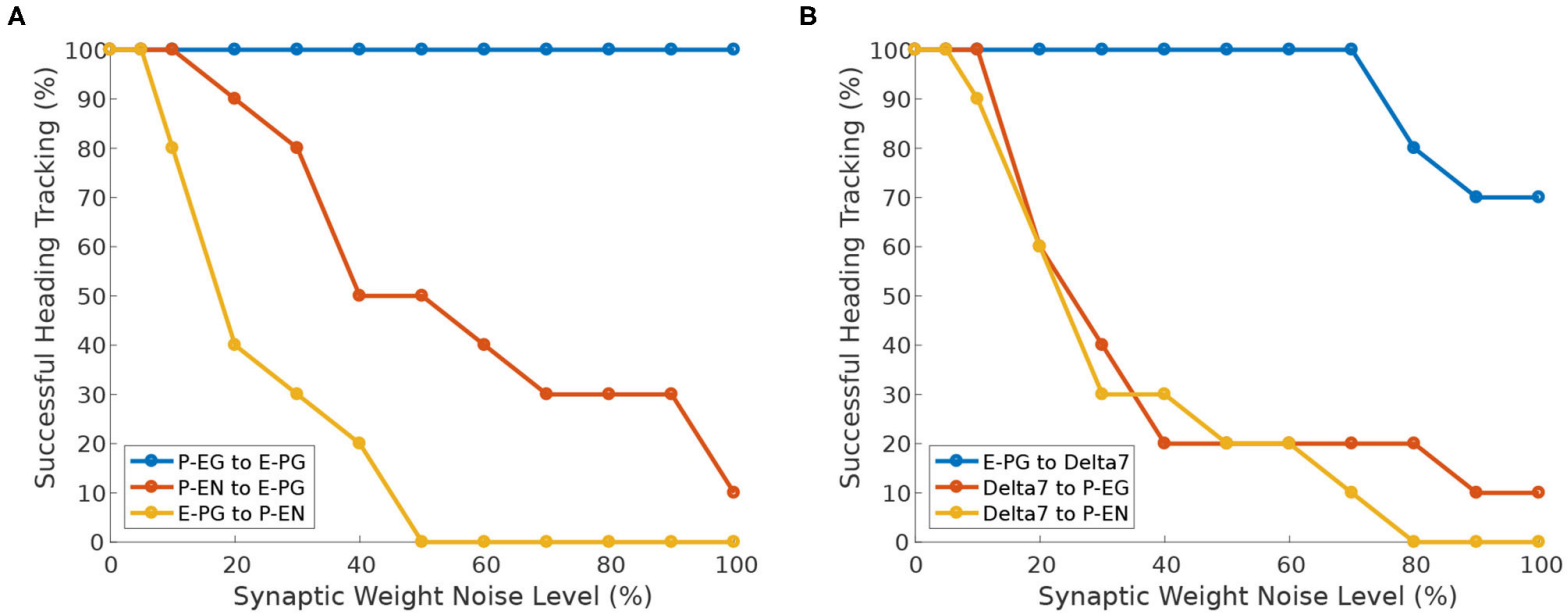
Effect of synaptic weights heterogeneity on heading tracking performance. The ability of the ring attractor to track the heading of a simulated robot, replicating the turns of a real fruit fly, as function of Gaussian noise added to synaptic weights for different classes of neuron synapses. **(A)** Effect of Gaussian noise on excitatory synapses. **(B)** Effect of Gaussian noise on synapses with Delta7 neurons on the presynaptic or postsynaptic side.

Next, we investigate the effect of heterogeneity introduced in different neuron synapses. While Pisokas et al. (2020) found that the P-EG neurons enhance the stability of the activity “bump,” in Figure 11A we see that the ability of the activity “bump” to successfully track the agent's heading, when the circuit is driven by heading stimulus, is unaffected by variation of the P-EG to E-PG synaptic weights. The ring attractor successfully tracks the agent's heading even if the P-EG neurons are completely silenced. This means that the P-EG neurons play an important role in maintaining a stable heading when no stimulus is provided but are not necessary when such a heading stimulus is present. Whether the inclusion of these neurons is justified in a particular ring attractor design would therefore depend on the operational environment and the agent's behavioral repertoire.

We can observe that the circuit is more sensitive to variations in the E-PG to P-EN synapses than variations of the P-EN to E-PG synapses (Figure 11A). The circuit is also sensitive to heterogeneity introduced in the inhibitory synapses from Delta7 neurons to P-EG and P-EN neurons since inhibition of excitatory neurons is an essential aspect of a ring attractor circuit for the emergence of an activity “bump” (Figure 11B).

However, the circuit is tolerant to variations of the input weights of Delta7 neurons (Figure 11B). This is because Delta7 neurons reciprocally synapse with each other, resulting in similar spiking activity in all of them due to averaging out the effect of synaptic weight variation.

Such insights drawn from observations about the ring attractor found in the brain of the fruit fly can be incorporated in building improved ring attractors with applications in robotics as well as in developing theoretical models. The ability to manipulate the circuit in robotic simulations can be used for testing hypotheses both at the neuron level and at the system level.

## 3. Discussion

The increasing availability of detail about neuronal structure, particularly in invertebrate brains, raises the possibility to simulate complete circuits. However, while directly implementing and simulating a biological neuronal circuit model allows us to understand the computation performed by it and to potentially derive its transfer function, it does not necessarily provide us with a real mechanistic understanding of its principle of operation and how its components interact. Reverse engineering the neuronal circuit can provide a real mechanistic understanding of the underlying principles of the computational structure. Such a mechanistic understanding is necessary for transfer to robotic technology because it would allow engineers to adapt the design to each application's particular needs.

An intriguing challenge was posed by Jonas and Kording (2017) who asked whether the tools and methods available to a neuroscientist would allow understanding of a microprocessor. Here, I have used reverse engineering techniques, borrowed from engineering, to reverse engineer the neuronal circuit that is encoding the head direction of the fruit fly. I derived the effective topological structure of the circuit and then determined (through optimization) the synaptic weights that would allow it to function as a ring attractor, mimicking the dynamics of the biological circuit. This illustrates that reverse engineering of a neuronal circuit with fewer than a hundred neurons is feasible.

It is worth noting that the circuit studied here, even though highly recurrent, has a regular structure that facilitates the systematic application of the presented procedure. It remains to be seen how this approach would need to be augmented in order to be tractably applied to circuits exhibiting less regularity. This highlights the need to develop tools that would assist the systematic analysis of larger neuronal circuits.

The availability of detailed neuron-level anatomical data and neuronal recordings from behaving animals in combination with computational simulations enabled the analysis and study of the circuit's organization and function. This level of detailed information is currently available for a few species, mainly insects. The fruit fly is one of these, allowing the application of the method to it. As data become available for more species and brain areas, we could have the opportunity to analyze more circuit structures and their function.

### 3.1. Assumptions and Simplifications

As any model, the present model is a simplification of the neuronal circuit found in the fruit fly brain; therefore, it is important to outline the assumptions made. The presented analysis is based on data collected using light microscopy (Wolff et al., 2015; Wolff and Rubin, 2018). Neurons with input and output synaptic terminals occupying the same volume were assumed to form synapses. Analysis of recently published electron microscopy data will allow more definite determination of synaptic connections between neurons and lead to more accurate models. Furthermore, all neurons in the model were assumed to have the same nominal biophysical property values. Of course, this will not be the case in the actual animals, but currently, there is no adequate data available about the biophysical properties of the individual neurons included in the model.

It was also assumed that Delta7 neurons have a uniform distribution of input terminals along the PB. Imaging of Delta7 neurons suggests a subtle variation of dendritic density along the PB, but it is yet unclear how this variation might relate to synaptic density and efficacy. Therefore, the simplifying assumption that the synaptic efficacy of Delta7 neurons along the PB is uniform was made. It was also assumed that neuronal terminals are clearly delineated and confined within the volumes of glomeruli and tiles. However, in some cases, stray terminals are known to sprout out to neighboring tiles of the EB (Turner-Evans et al., 2020). Such cross-innervation and interaction of EB volumes might have consequences for the connectivity of the circuit, potentially allowing a smoother transition of the activity “bump” between circuit octants. Future work will build upon the core circuit analyzed here and incorporate more circuit detail based on new electron microscopy data.

Occasionally neurons have mixed input and output terminals within the same volume. Given the uncertainty in the identification of the type of synaptic terminals, in those cases, the predominant terminal type was used. Furthermore, the synaptic weights of each type of synapse were assumed to be identical across neurons. This is not expected to be the case in actual fruit flies, especially for the neurons innervating tile T1 of the EB. This tile is innervated by twice the number of E-PG and P-EG neurons as other tiles; thus, some modulation of synaptic efficacy is expected in this volume in order to maintain a functional radial symmetry in the circuit. Such synaptic efficacy variation is suggested by the fact that the volumes of the innermost glomeruli of the PB are smaller than those of the other glomeruli (Wolff et al., 2015). Future functional connectivity studies will allow further investigation of this aspect.

It should also be noted that the ring topology of the resulting circuit alone does suggest but does not prove a ring attractor function. Here, the prior observation of neurobiological studies that the circuit maintains an activity “bump” that tracks the heading of the animal was used to impose constraints in the search for synaptic weights. For simplifying the computational complexity of the search for synaptic weights, it was assumed that all synapses between each neuron pair type are identical. Had the computational complexity of the search not been an issue, it would have been preferable to optimize all synaptic weights as independent parameters because that would have potentially revealed alternative weight configurations satisfying the objective function.

### 3.2. Nature as Inspiration for Theory and Engineering

The presented analysis method allowed us to unravel that the underlying head direction circuit has an eight-fold radial structure forming a closed ring (Pisokas et al., 2020). Without reverse engineering of the neuronal circuit, we would not have been able to see this underlying circuit structure, especially because, even though there are eight tiles in the EB, the PB has nine glomeruli in each hemisphere. As the connectivity results in a closed ring, it is an important aspect of the circuit, allowing the activity “bump” to move around the ring as the agent changes heading.

Combining reverse engineering with simulations enabled the identification of circuit elements that differ in several ways from the “canonical” ring attractor described in earlier theoretical models (e.g., Amari, 1977; Skaggs et al., 1995; Zhang, 1996). The P-EG neurons are a novel element in a ring attractor, forming local feedback loops within each octant of the circuit (reciprocal synapses between P-EG and E-PG neurons). These local reciprocal connections increase the tolerance of the circuit to structural noise in the synaptic weights, hence reducing the drift of the activity “bump” when no stimulus is provided (Pisokas et al., 2020); however, they are not important if the stimulus can be assumed at all times. This circuit component will be a useful trick in the toolkit of neuromorphic circuit designers.

Another difference from textbook ring attractor circuits revealed by the presented analysis method is that the P-EN neurons, instead of functioning as mere input neurons, are also part of the lateral excitation circuit (Pisokas et al., 2020). These neurons provide lateral excitation to their two nearest neighbors. P-EN neurons' dual function suggests a more efficient use of neuronal resources compared with typical ring attractor models that use separate sets of neurons for providing the lateral excitation and for rotating the activity “bump” around the ring in response to angular velocity input. The architecture of the ring attractor circuit found in the fruit fly and its differences from classical ring attractor models can inspire the design of novel ring attractor architectures with increased stability and efficient use of neuronal resources, both valuable aspects for applications in neuromorphic hardware and neurorobotics.

Reverse engineering gives us a mechanistic understanding of the underlying circuit, while computational simulations give us the tools to study the circuit's performance without having an analytical description of the model. Combined reverse engineering and computational simulations are tools that enable us to isolate and manipulate components of the neuronal structure in order to study their role in whole circuit. The mechanistic understanding of how the circuit components interact allows us to infer the circuit behavior under regimes beyond those explicitly tested with simulations. Combining these two tools allows us to obtain a deep understanding of neuronal circuits and enables us to learn their principles of operation.

Furthermore, the approach illustrated here shows that simulating the circuit as part of a robotic agent reveals aspects of the circuit's function that are masked when studying the circuit in isolation. For example, we saw that even if the ring attractor's response time is not sufficient for keeping up with fast turns of the agent, as long as the agent does not constantly turn faster than the circuit's response capability, and the heading stimulus is available, the ring attractor can readjust to the correct heading. We also saw that the P-EG neurons' presence, while essential for the stability of the activity “bump” when no stimulus is available, is not important to the circuit's function when a heading stimulus is available. These findings highlight the importance of characterizing neuronal circuits as part of behaving agents.

The studied circuit appears to be an effective means for an animal to internally track its orientation with respect to its surroundings and in insects appears to be a core component of a variety of navigation behaviors spanning from long-range migration to local path integration. The continued study of the detailed anatomy of the insect brain provides an exciting opportunity for the further unraveling of this circuit's function that evolved to support complex adaptive behavior.

## 4. Materials and Tools

### 4.1. Neuronal Nomenclature

Throughout this paper, I refer to neurons using their short names for brevity. The correspondence between the nomenclature used here and in the literature is shown in Table 1.

### 4.2. Neuron Model

The computational models and simulations were based on the source code of Kakaria and de Bivort (2017). The neurons were modeled as leaky integrate and fire units with refractory period. The membrane potential of each neuron was modeled by the differential Equation (1).

(1) $$\begin{array}{l} \frac{dV_{i}}{dt}=\frac{1}{C_{m}}\left(\frac{V_{0}-V_{i}}{R_{m}}+I_{i}+\overset{N}{\underset{j=1}{\sum }}M_{j,i}I_{j}\right) \end{array}$$

where *V*_*i*_ is the membrane potential of neuron *i*, *R*_*m*_ the membrane resistance, *C*_*m*_ the membrane capacitance, *I*_*i*_ the external input current to neuron *i*, *V*_0_ the resting potential, *M*_*j,i*_ the network connectivity matrix, *I*_*j*_ the output current of each neuron in the circuit and *N* is the number of neurons.

The neuron properties were set to the same values as those used by Kakaria and de Bivort (2017). These values are consistent with evidence from measurements in *D. melanogaster*. *C*_*m*_ was set to 2*nF* and *R*_*m*_ to 10*MΩ* for all neurons, assuming a surface area of 10^−3^*cm*^2^ (Gouwens and Wilson, 2009). The resting potential *V*_0_ was −52*mV* for all neurons (Rohrbough and Broadie, 2002; Sheeba et al., 2008) and the action potential threshold was −45 mV (Gouwens and Wilson, 2009). The action potential template was defined as (Kakaria and de Bivort, 2017):

(2) $$\begin{array}{l} V\left(t\right)=\\ \begin{array}{l} \;\{\begin{array}{c} V_{thr}+\left(V_{max}-V_{thr}\right)\frac{N\left(\frac{t_{tp}}{2},{\left(\frac{t_{AP}}{2}\right)}^{2}\right)-\alpha_{1}}{\beta_{1}},\text{ if }0\leq t<\frac{t_{AP}}{2}\\ V_{min}+\left(V_{max}-V_{min}\right)\frac{sin\left(\left(t-\frac{t_{AP}}{2}\right)\frac{2\pi }{t_{AP}}+\frac{\pi }{2}\right)+\gamma_{1}}{\delta_{1}},\text{ if }\frac{t_{AP}}{2}\leq t\leq t_{AP} \end{array} \end{array} \end{array}$$

When the membrane potential reached the threshold voltage *V*_*thr*_, the action potential template was inserted in the recorded voltage time series. *V*_*max*_ = 20 mV is the peak voltage (Rohrbough and Broadie, 2002) and *V*_*min*_ = −72 mV is the undershoot potential (Nagel et al., 2015). *t*_*AP*_ = 2 ms is the duration of the action potential (Gouwens and Wilson, 2009; Gaudry et al., 2012). $N\left(\mu ,\sigma^{2}\right)$ is a Gaussian function with mean μ and standard deviation σ. α_1_, β_1_, γ_1_, and δ_1_ are normalization parameters for scaling the range of the Gaussian and the sinusoidal to [0,1]. No other action potentials were allowed during the template duration in effect producing a refractory period.

The postsynaptic current generated by the action potential was modeled as (Kakaria and de Bivort, 2017):

(3) $$\begin{array}{l} I\left(t\right)=\{\begin{array}{cc} I_{PSC}\frac{sin\left(\frac{t\pi }{2}-\frac{\pi }{2}\right)+\alpha_{2}}{\beta_{2}}, & \text{if }0\leq t<2ms\\ I_{PSC}\frac{2^{-\left(t-2\right)/t_{PSC}}+\gamma_{2}}{\delta_{2}}, & \text{if }2ms\leq t\leq 2ms+7t_{PSC} \end{array} \end{array}$$

Excitatory and inhibitory postsynaptic currents were assumed to have the same magnitude but opposite signs. The parameters were set to *I*_*PSC*_ = 5 nA (Gaudry et al., 2012) and *t*_*PSC*_ = 5 ms (Gaudry et al., 2012). The postsynaptic current traces had duration 2*ms*+7*t*_*PSC*_ (2 ms of rise time plus 7*t*_*PSC*_ of decay time). α_2_, β_2_, γ_2_, and δ_2_ are normalization constants so that the range of the sinusoidal and exponential terms is [0,1]. Our simulation code was derived from the source code published by Kakaria and de Bivort (2017). The simulations were implemented in Matlab using Euler's method with a simulation time step of 10^−4^*s*. The source code is available at https://github.com/johnpi/Frontiers_Neurorobotics_Pisokas_2020.

### 4.3. Neuronal Projections and Connectivity Matrix

**Figure 12:**
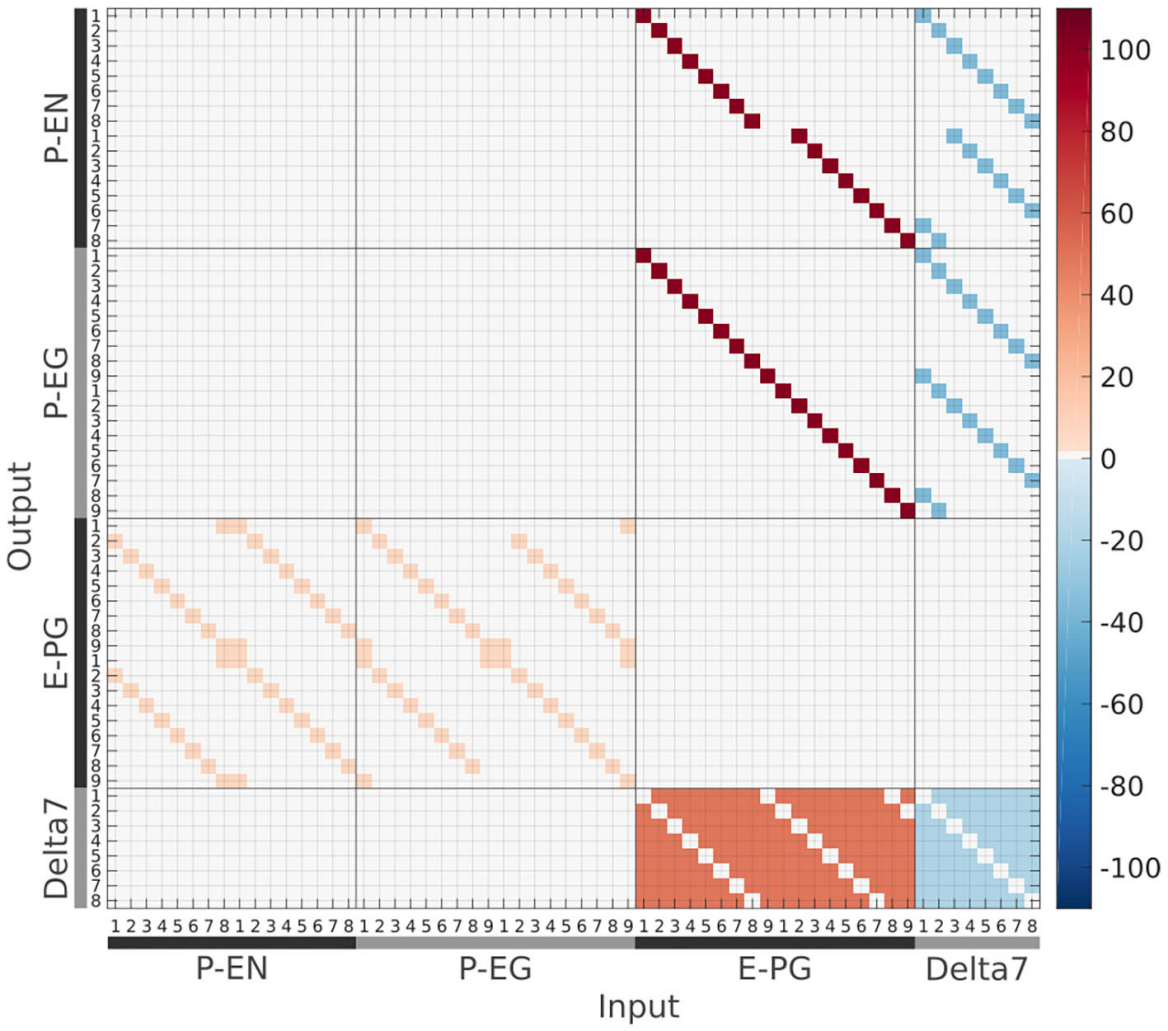
The connectivity matrix derived by the neuronal projection data of the fruit fly *Drosophila melanogaster* (Wolff et al., 2015; Wolff and Rubin, 2018). Synaptic weight is denoted by color in units of postsynaptic current equivalents.

The connectivity matrix of the circuit (Figure 12) has been inferred from anatomical data derived using light microscopy, with overlapping neuronal terminals assumed to form synapses between them (Wolff et al., 2015; Wolff and Rubin, 2018).

### 4.4. Stimuli

**Figure 13:**
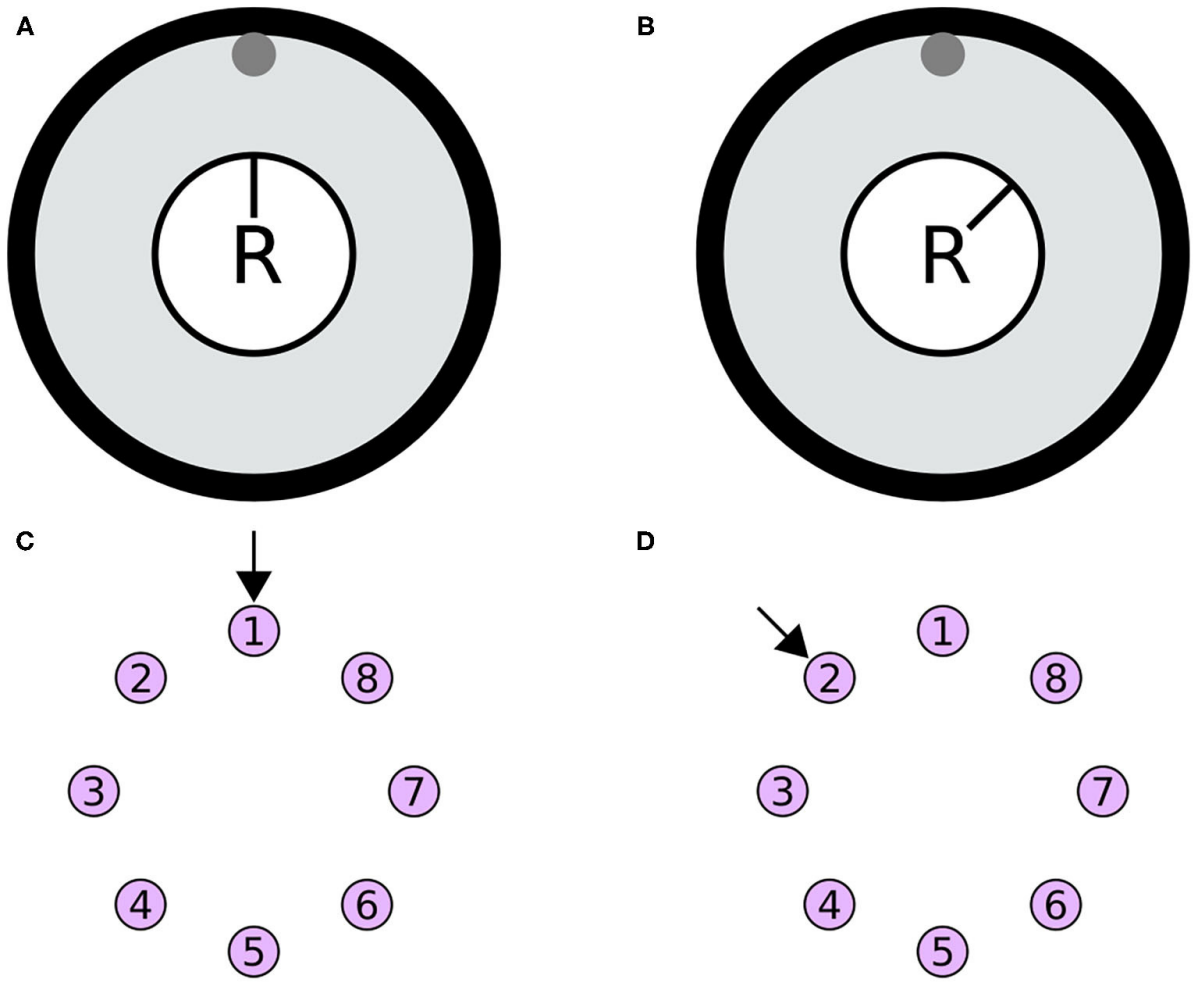
Simulated agent and its environment. **(A,B)** The simulated agent (R) is located in the middle of its environment (large black circle), with a visual cue (gray circle at the top of the environment). During the simulation the heading of the agent changes. **(C,D)** The azimuth of the visual cue with respect to the agent is mapped retinotopically to the E-PG neurons around the ring attractor. The azimuthal location is converted to a von Mises activity distribution and then to Poisson distributed spikes to stimulate the E-PG neurons. **(A,B)** Two poses of the agent with respect to the visual cue. **(C,D)** The corresponding stimulation of the E-PG neurons around the ring attractor.

The heading stimulus was provided as incoming spiking activity directly to the E-PG neurons. The heading, visual cue azimuth (Seelig and Jayaraman, 2015) around the animal or agent, was encoded as higher firing rates supplied to E-PG neurons at the corresponding location around the EB ring (Figure 13). The heading stimulus followed spatially a von Mises distribution with mean equal to the azimuth of the stimulus and full width at half maximum (FWHM) of approximately 90°. This was converted to Poisson distributed spike trains by sampling from a Poisson distribution. The background neuronal activity level was set to 5 impulses/s and the maximum stimulus activity was set to the peak level of activity of the E-PG neurons in the neuronal population.

### 4.5. Selection of Synaptic Weights

The free parameters of the model were the synaptic weights. The synaptic weights connecting each class of neurons were assumed to be identical, e.g., all E-PG to P-EN synapses had identical weights. Therefore, there was one free parameter for each synaptic class. To reduce the computational complexity during optimization, the synaptic weights of E-PG to P-EN and P-EG were identical as were the synaptic weights of Delta7 to P-EN and P-EG. This was the minimum set of independent synaptic weights that resulted in working ring attractors. The synaptic weights were modeled as the number of *I*_*PSC*_ unit equivalents flowing to the postsynaptic neuron per action potential.

The simulated annealing and particle swarm optimization algorithms were used to search for synaptic weights that resulted in working ring attractors (Matlab Optimization Toolbox “simulannealbnd” and “particleswarm” functions). The objective function optimized for solutions that produced an activity “bump” with a full width at half maximum (FWHM) of approximately 90° since this is the width that has been reported in fruit flies (Kim et al., 2017).

The objective function used to optimize the synaptic weights *w*_*i*_ was:

(4) $$\begin{array}{l} \underset{\text{w}}{\text{argmin}}\;4(\epsilon_{H1}(\text{w})+\epsilon_{H2}(\text{w}))+\epsilon_{W1}(\text{w})+\epsilon_{W2}(\text{w})+Np_{0}(\text{w})\\ \text{s.t. }\epsilon_{H1}(\text{w})=\frac{\left|H_{d}(t_{1})-H_{a}(\text{w},t_{1})\right|}{360^{{}^{\circ}}}\\ \;\epsilon_{H2}(\text{w})=\frac{\left|H_{d}(t_{2})-H_{a}(\text{w},t_{2})\right|}{360^{{}^{\circ}}}\\ \;\epsilon_{W1}(\text{w})=\frac{\left|90^{{}^{\circ}}-W_{a}(\text{w},t_{1})\right|}{360^{{}^{\circ}}}\\ \;\epsilon_{W2}(\text{w})=\frac{\left|90^{{}^{\circ}}-W_{a}(\text{w},t_{2})\right|}{360^{{}^{\circ}}}\\ \;p_{0}(\text{w})=\frac{1}{N}\sum_{i=1}^{N}{\left(e^{-|w_{i}|}\right)}^{2}\\ \begin{array}{l} \;0\leq w_{1}\leq 100\\ \;0\leq w_{2}\leq 100\\ \;0\leq w_{3}\leq 100\\ \;-100\leq w_{4}\leq 0\\ \;-100\leq w_{5}\leq 0 \end{array} \end{array}$$

Where ϵ_*H*1_, ϵ_*H*2_, ϵ_*W*1_, and ϵ_*W*2_ are the error factors measured as deviations from the desired values. *H*_*d*_(*t*) is the desired activity “bump” heading at time *t*, while *H*_*a*_(**w**, *t*) is the actual activity “bump” heading at time *t* given a model with synaptic weights **w**. *W*_*a*_(**w**, *t*) is the actual width of the activity “bump” at time *t* (measured as the full width at half maximum). *p*_0_ is used to penalize synaptic weights that are too close to 0 and *N* is the number of synaptic weights *w*_*i*_. The constraints in 4 specify that the synapses with Delta7 neurons at their presynaptic side are inhibitory (negative) and all others are excitatory (positive). Excitatory synaptic weights were initialized with value 0.01 and inhibitory synaptic weights with value −0.01. During optimization, the model was simulated to search the space of synaptic weights. The objective function was used to optimize the synaptic weights separately for the two models, the fruit fly model and the one without P-EG neurons. The optimized synaptic weight sets were manually tested to verify the results.

### 4.6. Sensitivity Analysis

For the sensitivity analysis, white Gaussian noise was added to the synaptic weights, using the formula

(5) $$\begin{array}{l} w_{i}=w_{nominal}+\frac{x}{100}w_{nominal}\epsilon ,\\ \;\epsilon \sim N\left(\mu ,\sigma^{2}\right) \end{array}$$

where *w*_*i*_ is the resulting noisy value of weight *i*. *i* = {1, 2, …, *M*} and *M* is the number of weights. *w*_*nominal*_ is the nominal value of the weight, *x* ∈ [0, 100] is the percentage of noise to be added to the nominal value, ϵ is a random variable sampled from the Gaussian distribution with μ = 0 and σ^2^ = 1. The number of successful trials was counted in each condition. The criterion for a successful trial was that the activity “bump” tracked the stimulus heading with an error of <±10° for more than 50% of stimulus duration.

## Data Availability Statement

Publicly available datasets were analyzed in this study. This data can be found at: https://doi.org/10.1002/cne.24512.

## Author Contributions

IP conceptualized and developed the method for deriving the effective circuit and contributed to the experimental design, software, validation of results, statistical analysis, visualizations, and manuscript writing.

## Conflict of Interest

The author declares that the research was conducted in the absence of any commercial or financial relationships that could be construed as a potential conflict of interest.

## References

[B1] Amari S. (1977). Dynamics of pattern formation in lateral-inhibition type neural fields. Biol. Cybern. 27, 77–87. 10.1007/BF00337259 911931

[B2] Cichy R. M. Khosla A. Pantazis D. Torralba A. Oliva A. (2016). Comparison of deep neural networks to spatio-temporal cortical dynamics of human visual object recognition reveals hierarchical correspondence. Sci. Rep. 6, 1–13. 10.1038/srep27755 27282108 PMC4901271

[B3] Cope A. J. Sabo C. Vasilaki E. Barron A. B. Marshall J. A. (2017). A computational model of the integration of landmarks and motion in the insect central complex. PLoS ONE 12:e0172325. 10.1371/journal.pone.0172325 28241061 PMC5328262

[B4] Gaudry Q. Hong E. J. Kain J. de Bivort B. L. Wilson R. I. (2012). Asymmetric neurotransmitter release enables rapid odour lateralization in Drosophila. Nature 493, 424–428. 10.1038/nature11747 23263180 PMC3590906

[B5] Giraldo Y. M. Leitch K. J. Ros I. G. Warren T. L. Weir P. T. Dickinson M. H. (2018). Sun navigation requires compass neurons in Drosophila. Curr. Biol. 28, 2845–2852.e4. 10.1016/j.cub.2018.07.002 30174187 PMC7301569

[B6] Gouwens N. W. Wilson R. I. (2009). Signal propagation in Drosophila central neurons. J. Neurosci. 29, 6239–6249. 10.1523/JNEUROSCI.0764-09.2009 19439602 PMC2709801

[B7] Green J. Adachi A. Shah K. K. Hirokawa J. D. Magani P. S. Maimon G. (2017). A neural circuit architecture for angular integration in Drosophila. Nature 546, 101–106. 10.1038/nature22343 28538731 PMC6320684

[B8] Green J. Maimon G. (2018). Building a heading signal from anatomically defined neuron types in the Drosophila central complex. Curr. Opin. Neurobiol. 52, 156–164. 10.1016/j.conb.2018.06.010 30029143 PMC6320682

[B9] Homberg U. Heinze S. Pfeiffer K. Kinoshita M. El Jundi B. (2011). Central neural coding of sky polarization in insects. Philos. Trans. R. Soc. B Biol. Sci. 366, 680–687. 10.1098/rstb.2010.0199 21282171 PMC3049008

[B10] Jonas E. Kording K. P. (2017). Could a neuroscientist understand a microprocessor? PLoS Comput. Biol. 13, 1–24. 10.1371/journal.pcbi.1005268 28081141 PMC5230747

[B11] Kakaria K. S. de Bivort B. L. (2017). Ring attractor dynamics emerge from a spiking model of the entire protocerebral bridge. Front. Behav. Neurosci. 11:8. 10.3389/fnbeh.2017.00008 28261066 PMC5306390

[B12] Kim S. S. Rouault H. Druckmann S. Jayaraman V. (2017). Ring attractor dynamics in the Drosophila central brain. Science 356, 849–853. 10.1126/science.aal4835 28473639

[B13] Liu G. Seiler H. Wen A. Zars T. Ito K. Wolf R.. (2006). Distinct memory traces for two visual features in the Drosophila brain. Nature 439, 551–556. 10.1038/nature04381 16452971

[B14] Martin J. P. Guo P. Mu L. Harley C. M. Ritzmann R. E. (2015). Central-complex control of movement in the freely walking cockroach. Curr. Biol. 25, 2795–2803. 10.1016/j.cub.2015.09.044 26592340

[B15] Nagel K. I. Hong E. J. Wilson R. I. (2015). Synaptic and circuit mechanisms promoting broadband transmission of olfactory stimulus dynamics. Nat. Neurosci. 18, 56–65. 10.1038/nn.3895 25485755 PMC4289142

[B16] Neuser K. Triphan T. Mronz M. Poeck B. Strauss R. (2008). Analysis of a spatial orientation memory in Drosophila. Nature 453, 1244–1247. 10.1038/nature07003 18509336

[B17] Ofstad T. A. Zuker C. S. Reiser M. B. (2011). Visual place learning in Drosophila melanogaster. Nature 474, 204–209. 10.1038/nature10131 21654803 PMC3169673

[B18] Pisokas I. Heinze S. Webb B. (2020). The head direction circuit of two insect species. eLife. 9:e53985 10.7554/eLife.53985.sa2 32628112 PMC7419142

[B19] Rekoff M. G. (1985). On reverse engineering. IEEE Trans. Syst. Man Cybern. 15, 244–252. 10.1109/TSMC.1985.6313354

[B20] Ritzmann R. E. Harley C. M. Daltorio K. A. Tietz B. R. Pollack A. J. Bender J. A.. (2012). Deciding which way to go: how do insects alter movements to negotiate barriers? Front. Neurosci. 6:97. 10.3389/fnins.2012.00097 22783160 PMC3390555

[B21] Rohrbough J. Broadie K. (2002). Electrophysiological analysis of synaptic transmission in central neurons of Drosophila larvae. J. Neurophysiol. 88, 847–860. 10.1152/jn.2002.88.2.847 12163536

[B22] Seelig J. D. Jayaraman V. (2015). Neural dynamics for landmark orientation and angular path integration. Nature 521, 186–191. 10.1038/nature14446 25971509 PMC4704792

[B23] Sheeba V. Gu H. Sharma V. K. O'Dowd D. K. Holmes T. C. (2008). Circadian- and light-dependent regulation of resting membrane potential and spontaneous action potential firing of drosophila circadian pacemaker neurons. J. Neurophysiol. 99, 976–988. 10.1152/jn.00930.2007 18077664 PMC2692874

[B24] Skaggs W. E. Knierim J. J. Kudrimoti H. S. McNaughton B. L. (1995). A model of the neural basis of the rat's sense of direction. Adv. Neural Inform. Process. Syst. 7, 173–80. 11539168

[B25] Stone T. Webb B. Adden A. Weddig N. B. Honkanen A. Templin R.. (2017). An anatomically constrained model for path integration in the bee brain. Curr. Biol. 27, 3069–3085.e11. 10.1016/j.cub.2017.08.052 28988858 PMC6196076

[B26] Strauss R. (2002). The central complex and the genetic dissection of locomotor behaviour. Curr. Opin. Neurobiol. 12, 633–638. 10.1016/S0959-4388(02)00385-9 12490252

[B27] Su T. S. Lee W. J. Huang Y. C. Wang C. T. Lo C. C. (2017). Coupled symmetric and asymmetric circuits underlying spatial orientation in fruit flies. Nat. Commun. 8. 10.1038/s41467-017-00191-6 28747622 PMC5529380

[B28] Tammero L. F. Dickinson M. H. (2002). The influence of visual landscape on the free flight behavior of the fruit fly Drosophila melanogaster. J. Exp. Biol. 205(Pt 3), 327–343. 11854370 10.1242/jeb.205.3.327

[B29] Taube J. Muller R. Ranck J. (1990). Head-direction cells recorded from the postsubiculum in freely moving rats. I. Description and quantitative analysis. J. Neurosci. 10, 420–435. 10.1523/JNEUROSCI.10-02-00420.1990 2303851 PMC6570151

[B30] Triphan T. Poeck B. Neuser K. Strauss R. (2010). Visual targeting of motor actions in climbing Drosophila. Curr. Biol. 20, 663–668. 10.1016/j.cub.2010.02.055 20346674

[B31] Turner-Evans D. Wegener S. Rouault H. Franconville R. Wolff T. Seelig J. D.. (2017). Angular velocity integration in a fly heading circuit. eLife 6, 2112–2126. 10.7554/eLife.23496 28530551 PMC5440168

[B32] Turner-Evans D. B. Jensen K. Ali S. Paterson T. Sheridan A. Ray R. P. Wolff T.. (2020). The neuroanatomical ultrastructure and function of a biological ring attractor. Neuron 108, 145–163. 10.1016/j.neuron.2020.08.006 32916090 PMC8356802

[B33] Varga A. G. Kathman N. D. Martin J. P. Guo P. Ritzmann R. E. (2017). Spatial navigation and the central complex: sensory acquisition, orientation, and motor control. Front. Behav. Neurosci. 11:4. 10.3389/fnbeh.2017.00004 28174527 PMC5258693

[B34] Wolff T. Iyer N. A. Rubin G. M. (2015). Neuroarchitecture and neuroanatomy of the Drosophila central complex: a GAL4-based dissection of protocerebral bridge neurons and circuits. J. Compar. Neurol. 523, 997–1037. 10.1002/cne.23705 25380328 PMC4407839

[B35] Wolff T. Rubin G. M. (2018). Neuroarchitecture of the Drosophila central complex: a catalog of nodulus and asymmetrical body neurons and a revision of the protocerebral bridge catalog. J. Compar. Neurol. 526, 2585–2611. 10.1002/cne.24512 30084503 PMC6283239

[B36] Yamins D. L. DiCarlo J. J. (2016). Using goal-driven deep learning models to understand sensory cortex. Nat. Neurosci. 19, 356–365. 10.1038/nn.4244 26906502

[B37] Yamins D. L. Hong H. Cadieu C. F. Solomon E. A. Seibert D. DiCarlo J. J. (2014). Performance-optimized hierarchical models predict neural responses in higher visual cortex. Proc. Natl. Acad. Sci. U.S.A. 111, 8619–8624. 10.1073/pnas.1403112111 24812127 PMC4060707

[B38] Young J. M. Armstrong J. D. (2010). Structure of the adult central complex in Drosophila: organization of distinct neuronal subsets. J. Compar. Neurol. 518, 1500–1524. 10.1002/cne.22284 20187142

[B39] Zhang K. (1996). Representation of spatial orientation by the intrinsic dynamics of the head-direction cell ensemble: a theory. J. Neurosci. 16, 2112–2126. 10.1523/JNEUROSCI.16-06-02112.1996 8604055 PMC6578512

